# Gynecological Laparoscopic Surgeries under Spinal Anesthesia: Benefits and Challenges

**DOI:** 10.3390/jpm14060633

**Published:** 2024-06-14

**Authors:** Attila L. Major, Kudrat Jumaniyazov, Ruslan Jabbarov, Mehdi Razzaghi, Ivanna Mayboroda

**Affiliations:** 1 Femina Gynecology Centre, CH-1205 Geneva, Switzerland; 2 Faculty of Sciences and Medicine, University of Fribourg, CH-1700 Fribourg, Switzerland; 3 Department of Gynecology and Obstetrics, Urgench Branch of Tashkent Medical Academy, Urgench 220100, Uzbekistan; 4 Department of Anesthesiology, Urgench Branch of Tashkent Medical Academy, Urgench 220100, Uzbekistan; 5 Department of Mathematics, Computer Science, and Digital Forensics, Bloomsburg University, Bloomsburg, PA 17815, USA; 6 Department of Gynecology and Obstetrics, Regional Hospital of Yverdon-les-Bains, CH-1400 Yverdon, Switzerland

**Keywords:** spinal anesthesia, laparoscopic surgeries, gynecology, mini-invasive operations

## Abstract

Objective: This prospective study investigated the feasibility of performing laparoscopic pelvic surgery under spinal anesthesia and analyzed the intraoperative side effects, like pain, nausea, and vomitus, of 915 patients. Methods: The implementation and performance of laparoscopic surgery under local anesthesia on 915 patients (out of a total of 3212 who underwent laparoscopic pelvic surgery under spinal anesthesia) were analyzed in relation to BMI (body mass index), obesity, pain during surgery, amount of intraperitoneal mmHg CO_2_ gas pressure, and surgical complications. Results: BMI > 30, intra-abdominal adhesions, increased duration of the operation, bleeding, and increased intraperitoneal CO_2_ pressure were statistically significant as the main causes of pain during laparoscopic surgery under spinal anesthesia. Underweight patients, on the other hand, had less pain when intra-abdominal pressure increased compared to those of normal weight. The appearance of pain, nausea, and vomitus occurred in 10.3% of patients, and these events were easy to manage and treat. They did not affect the surgeon’s work or the course of the operation. Conclusions: In light of these observations, we are proposing spinal anesthesia for laparoscopic surgery as the first choice in patients who have no contraindications. To the best of our knowledge, this clinical study constitutes the largest clinical observation and dataset concerning spinal anesthesia in laparoscopic pelvic surgery. Trial registration: ISRCTN38987, 10 December 2019.

## 1. Introduction

In global practice, general anesthesia is considered a common method for conducting laparoscopic abdominal surgeries. Spinal anesthesia is assumed as the next alternative when the patient has contraindications to total anesthesia. The authors discuss their experience of applying spinal anesthesia as the first choice for laparoscopy surgery, clinically proving that it is the best option for anesthesia. Over 3212 laparoscopic gynecology surgeries were conducted in Urgench (Uzbekistan) with spinal anesthesia to observe the benefits and challenges of this minimally invasive operation.

In 2020, there were over 40,000 birth deliveries in the Khorezm region (which has a population of 1.77 million), and 8795 patients had a cesarean section (Table 1). The majority (98.1%) of patients had epidural (spinal) anesthesia [1]. Regional anesthesia (RA) is a common practice in the Khorezm region of Uzbekistan and anesthesiologists widely use this method with outstanding outcomes. Regional anesthesia for cesarean sections, compared to general anesthesia (GA), provides less maternal mortality, the possibility of using fewer drugs, more direct practice of childbirth [2,3], and the opportunity to diminish blood loss while ensuring outstanding postoperative pain control [4]. 

For laparoscopic surgery, however, peridural anesthesia is insufficient. The relaxation of the abdominal muscles by spinal anesthesia, enabling the insufflation of the peritoneal cavity sufficiently, has to be performed. In order to make the gynecological organs, covered by the intestines, visible, the Trendelenburg position is needed [5]. However, the induction of spinal anesthesia in the Trendelenburg position by inexperienced personnel can be dangerous because anesthesia like bupivacaine reaching the thoracic spinal canal can block the correct functioning of the respiratory organs and consequently arrest spontaneous breathing. The other reason why general anesthesia is still preferred for laparoscopy in gynecology is the need for a sufficiently high intra-abdominal pressure of CO_2_ gas to blow the abdominal cavity in order to achieve a good endoscopic view, which results in the risk of unwanted surgical side effects. On one side, loco-regional anesthesia may cause discomfort and pain to the patient; on the other side, most surgeons are not used to performing surgery in low CO_2_ pressure, which is the condition in which most patients feel comfortable. 

To carry out spinal anesthesia in the Trendelenburg position, it is important to have physical knowledge of the role of hyperbaric solutions in anesthesia medicine [6] and to have trained personnel [7]. Such anesthetics include the 0.5% hyperbaric solution of bupivacaine hydrochloride (Longocain Heavy). When a hyperbaric solution is injected into the cerebrospinal fluid, it sinks and drops below the puncture site, causing anesthesia in the corresponding segment [8,9,10]. If the puncture is performed in the L2–L3 region and the patient is in the supine position, the hyperbaric solution, with anesthesia like bupivacaine, flows from the apex of the lumbar lordosis in both directions. When the patient is in a lateral recumbent position, the anesthesia effect will be on the corresponding side [8,9]. This also means that when the body is tilted to the Trendelenburg position, the anesthetic medicine spreads unhindered in the cranial direction to the thoracic segments and the nerves innervating the respiratory organs [10,11]. However, when performing the puncture in a sitting position and leaving the patient in this position for a while [12], a classic “saddle” anesthetic block develops [5,13]. By applying it correctly, anesthesia in hyperbaric solution is fixed at the correct side, enabling laparoscopic surgery without affecting the respiratory organs. 

The Trendelenburg position also has advantages. Among other factors, it improves venous return and harmonizes blood pressure [5,14]. There are several key factors that discourage the use of laparoscopic surgery under spinal anesthesia. Firstly, the surgical and anesthesiology teams may lack the necessary expertise to perform both anesthesia and surgery in the Trendelenburg position, which is required for gynecological laparoscopic procedures. Additionally, the unfamiliar and potentially challenging environment of the operating theater can contribute to patient anxiety, mainly due to a lack of confidence if the patient is not well prepared by the medical team. While the occurrence of nausea, vomiting, and discomfort during the surgical intervention is a possible inconvenience, it can be effectively managed with proper care. Notably, the conscious state of the patient under spinal anesthesia eliminates the risk of aspiration into the lungs in case of vomiting. It is the responsibility of the anesthetist and surgeon to provide clear explanations and reassurance to address any concerns related to nausea and vomiting. Importantly, opting for laparoscopic surgery under spinal anesthesia offers distinct advantages, such as accelerated postoperative recovery, reduced incidence of postoperative nausea and vomiting, and a decreased requirement for analgesic medications. The intensity of pain and discomfort is directly related to the pressure of the pneumoperitoneum [5]. It can be managed by reducing intra-abdominal pressure.

According to several retrospective research studies on clinical databases, endoscopic surgeries performed under neuraxial anesthesia, such as spinal anesthesia, have been associated with reduced patient mortality and major morbidity, including pulmonary complications and transfusion requirements, compared to classical surgeries performed under general anesthesia [4,11]. The same was also described for laparoscopic surgeries [15]. Note that in difficult times such as the COVID pandemic, there was not enough anesthetic material, drugs, and personnel available for surgical procedures under general anesthesia, which can be prevented or at least reduced with more interventions in spinal and epidural anesthesia [16]. Another advantage of the regional anesthesia technique has been shown to reduce the length of hospital stay, which is important in times of catastrophes [17]. One of the advantages of local regional anesthesia (LRA) is better postoperative pain control, which has been linked to spinal drug injection in the LRA group [5,17]. Patients who receive general anesthesia often experience initial pain upon regaining consciousness postoperatively. Comparisons between the benefits of spinal anesthesia and general anesthesia for postoperative pain control showed statistical significance at subsequent testing periods, such as 8, 12, 24, and 48 h after surgery [17]. Patients who received spinal anesthesia did not require intravenous opioid administration, and they achieved a quick return of intestinal motility and independent ambulation. It also shortened the period of urinary catheterization and decreased the infection risk. Avoiding extended bed rest, which can result in paralytic ileus, muscle soreness, and weariness, also encourages better postoperative pain management.

Intra- and postoperative advantages of spinal anesthesia combined with LRA were observed, including significantly lower pain scores in the postoperative period compared to general anesthesia [17]. A statistically significant difference of 6 points in visual analog scale pain scoring (VAS), a validated subjective measurement of acute and chronic pain, was recorded one hour after surgery, as reported in a study [17]. Our study and these findings support increasing and better defining the use of spinal anesthesia and LRA for laparoscopic surgeries in the future.

## 2. Materials and Methods

### 2.1. Study Group

A total of 3212 women participated in the prospective study and had laparoscopic surgery of the pelvis using spinal anesthesia in Urgench City of Uzbekistan from 9 January 2019 to 31 January 2024 [5,18]. The following parameters were recorded for each patient: Indication for surgery; BMI (body mass index); parity; the presence of adhesive disease; volume and duration of the intervention; pain before, during, and after surgery; localization of pain; amount of intraperitoneal mmHg CO_2_ gas pressure; the quantity of blood loss during the intervention, etc. The pain was recorded by questioning the patients and was scored from 1–10: mild 1–4, moderate 5–6, severe 7–8 and very strong 9–10. A total of 915 of these interventions were analyzed statistically. Patients ranged in age from 25 to 50 years. The consent from each patient was asked prior to the operation. A quality questionnaire with a pain score was performed before, during, and after surgery. Data of 915 patients operated on until 6 March 2021 using spinal anesthesia were entered into the database and analyzed.

### 2.2. Technique of Spinal Anesthesia

To perform spinal anesthesia in the Trendelenburg position, it is important to have physical knowledge of anesthesia medicine in a hyperbaric solution and to have trained personnel. Such anesthetics include a 0.5% hyperbaric solution of bupivacaine hydrochloride (Longocain Heavy). When a hyperbaric solution is injected into the cerebrospinal fluid, it sinks and drops below the puncture site, causing anesthesia in the corresponding segment. If the puncture is administered in the L2–L3 region and the patient is in the supine position, the hyperbaric solution with anesthesia, like bupivacaine, flows from the apex of the lumbar lordosis in both directions. When the patient is in a lateral recumbent position, the anesthesia effect will be on the corresponding side. This also means that when the body is tilted to a Trendelenburg position, the anesthetic medicine spreads unhindered in the cranial direction to the thoracic segments and to the nerves, innervating the breathing organs. On the other hand, performing the puncture in a sitting position and leaving the patient in this position for a while, a classic “saddle” anesthetic block develops. 

For anesthesia of the abdomen, the anesthetic is injected into the spinal canal of L2–L3 and the patient is in a sedentary position. Immediately after, the patient is slowly positioned into a Trendelenburg position of minus 15 degrees. This way, the anesthetic moves gently in the cranial direction. At this time, special care is required since too strong a flow of the anesthetic in the direction of the thoracic segments of the spinal canal can cause blockage of breathing and apnea. By holding the patient in a slight Trendelenburg position, the anesthesia moves in the direction of the xiphoid. After placement of the spinal anesthesia, the patient is moved from the Trendelenburg to a horizontal position. Then, the level of anesthesia on the abdomen is evaluated by touching the skin (Algometry). The level between the abdomen and the thorax should not be exceeded. When the anesthetist detects anesthesia effects above the umbilicus or the beginning of bradycardia is observed, the patient is placed in a slightly anti-Trendelenburg position so that the anesthetic moves back in the caudal direction until the anesthetic is fixed. This lasts a few minutes. By this procedure, respiratory arrest is prevented with certainty. Immediately after the placement of the spinal anesthesia, oxygen is routinely required for all patients because of the danger of cerebral hypoxia as a cause of hypotension. It is very important to follow the monitoring of blood pressure, pulse, and oxygen at this moment. An epigastric sonde was placed.

To prevent complications due to SA, the authors paid serious attention to comorbid diseases of patients and contraindications of SA, and discussed with other specialists the relative contraindications of SA. By using a special technique as well as preoperative monitoring and intraoperative vigilance, there is a very low risk of SA for patients. In the rare occurrence of when a problem with SA might happen, intubation material and devices for general anesthesia must be available for immediate use. 

In the case of tachycardia and low blood pressure, Atropine is administered. The causes of nausea and vomiting are low blood pressure and high intra-abdominal pressure. Nausea and vomitus often occur at the beginning of surgery and can be prevented by Atropine acting as a cholemimetic and blocking the secretion of the gastrointestinal tract and secretion of saliva. Nausea and vomitus, associated and induced by hypotension, are prevented by the perfusion of physiological solution and by the administration of the sympathomimetic and vasopressor phenylephrine (Mesaton) to increase blood pressure. The use of measures for the prevention and treatment of hypotension, as well as for the prevention of spinal blockade, included the administration of an infusion of crystalloids and/or colloids, wrapping the lower limbs with compression stockings or bandages, the introduction of an optimal dose of local anesthetic, a special technique of bending the body for the spinal injection, and the administration of inotropes and vasopressors. 

In case nausea and vomitus happen, the operation table with the patient is returned to the horizontal position, the head of the patient is turned to the side, and the patient is given the opportunity to vomit. A maximum of 5 min is enough in order to continue surgery in the Trendelenburg position. There is no danger because the patient is conscious. After that, she is removed from the Trendelenburg position in order to continue the operation. Usually, there is a single event of vomitus during the operation. The vomit is a secretion of the gastrointestinal tract, which has accumulated in the lumen of the stomach and intestines already before the administration of Atropine. At this point, Atropine prevents only nausea and vomiting. This is the reason why most nausea and vomiting happen at the beginning of surgery. The reason why it happens only once is that during the operation, the Atropine hinders the production of more saliva and gastric secretion.

During and after surgery, high intra-abdominal pressure can be present because of the irritation of the peritoneum in the region of the diaphragm, where there is no anesthesia. This occurs mainly for large and long surgeries, which are associated with pain induced by adhesions and bleeding.

### 2.3. Special Technique of Laparoscopic Surgery under Spinal Anesthesia

The patient’s position depends on the location to be operated, such as the Trendelenburg position for the pelvic organs in gynecological procedures. Inducing spinal anesthesia by inexperienced personnel can be dangerous because bupivacaine and other anesthesia in the spinal canal can block breathing. By using the described technique below and by monitoring and vigilance, there is a very low risk for the patient. In rare cases, it may nevertheless happen; intubation material and devices for general anesthesia must be available for immediate use. Among our 3212 patients with laparoscopic surgery in spinal anesthesia, there was not one case with respiratory arrest. Before moving the patient to the Trendelenburg position, the patient is held for 10 min in the horizontal position to fix anesthetic medicine in the spinal canal. Once the medicine is fixed, there is no longer a risk that anesthesia of the breathing organs will occur by moving the patient to the Trendelenburg position. The advantage of Trendelenburg is that it improves venous return and harmonizes blood pressure.

An intraumbilical incision was performed, and at both sides of the incision, the skin was elevated with clamps. A Veress needle was introduced intraperitoneally to enable CO_2_ insufflation with low pressure. A 10 mm Trocar was inserted in the umbilical area, and 2 to 3 five mm trocars were inserted in the lower abdomen. The same procedure was performed even in patients after a cesarean section. To evacuate excised tissue, the lateral incision of the 5 mm trocar was extended if necessary.

The intensity of pain and discomfort is directly related to the pressure of the pneumoperitoneum. It can be managed by reducing intra-abdominal pressure. In our experience of 915 patients, a good compromise is 8 mmHg or lower; sometimes 10 mmHg is also well endured. In the beginning, the surgeon has to take time to adapt to working with such low pressures. Such an adaptation, however, can occur quickly if the surgeon is motivated, and this can occur without respiration problems in the patient by doing such surgery with a pneumoperitoneum of 8 mmHg and in a Trendelenburg position of 30–45°. To have good laparoscopic visibility and not be disturbed by the intestines, some precautions are important: (1) a good preoperative preparation of the bowel, (2) a Trendelenburg position of 30–45°, and (3) manipulation of the uterus by a vaginal or laparoscopic device.

All laparoscopic operations were performed under spinal anesthesia. 

Before moving the patient to the Trendelenburg position, the patient is held for 10 min in the horizontal position to fix anesthetic medicine in the spinal canal [5,18]. Once the medicine is fixed, there is no longer a risk that anesthesia of the breathing organs will occur by moving the patient to the Trendelenburg position. To study the factors influencing the occurrence of pain, an analysis was carried out depending on the abdominal status, the duration of the operation, the volume of blood loss, the presence of nausea and vomiting during the operation, and the volume of intra-abdominal pressure during the operation.

### 2.4. Describing as a Statistical Model 

Factors influencing the occurrence of pain were further analyzed using a statistical model known as ‘analysis of covariance’ or ANCOVA for short. This is a method that allows the determination of the relationship between two variables. Here, we used the occurrence of pain and obesity while controlling for a third variable—the amount of CO_2_ gas used to relieve pain. The third variable is commonly referred to as a ‘covariate’. The methodology was first used in the 1930s in agricultural applications, but since then, it has been widely used in many other fields. Analysis of covariance is known to increase the statistical power of the significance test [19,20]. 

Note that in our analysis, the ‘occurrence of pain’, called the response variable, is dichotomous in nature. That is, a patient either experiences pain or is free of pain. Generalization to cases where there is a degree of pain, i.e., the response variable is ordinal, is possible. Therefore, the statistical model regressing the occurrence of pain on the explanatory variables ‘amount of utilized CO_2_ gas’ and ‘obesity’ is based on the so-called logistic regression [21]. The model assumes that the odds of having pain to being free of pain on the logarithmic scale, called the logit, is a linear function of the explanatory variables.

## 3. Results

All patients who underwent surgery, depending on the presence of pain, were divided into two groups (Table 2). The first group was women who did not feel pain during the operation according to the VAS scale (the degree of pain intensity according to VAS) as follows:

(1) No pain—0;
(2) Weak—up to 40%;
(3) Moderate—40–70%;
(4) Severe—more than 70%;
(5) Unbearable—100%

Consequently, 847 (92.5%) patients out of 915 women varied in this range (Table 3). The second group consisted of women who had mild or moderate pain during surgery, of which were 68 (7.5%) patients. To study the factors influencing the occurrence of pain, an analysis was carried out depending on age, body mass index, obesity, parity, and concomitant diseases. A significant difference between the groups was found in the one with patients who had adhesive disease and who underwent dissection of adhesions during surgery. In the first group, women with adhesive disease accounted for 18 (2.1%), and in the second group, 6 (8.8%). In addition, a statistically significant difference between the groups was defined as individuals with a body mass index exceeding 30—body mass index—˃30. In the first group, obese women accounted for 63 (7.44%), and in the second group, 13 (19.12%), respectively. When analyzing other factors influencing the onset of pain, no statistically significant factors were found between the groups.

The results of the statistical analysis showed that with such indicators as a fourth degree of abdominal status, the duration of the operation is more than 60 min. In addition, blood loss over 50 mL, body mass index over 30, and intra-abdominal pressure over 5 mm are statistically significant indicators that play a fundamental role in the development of moderate or mild pain during surgery (*p* ≤ 0.05; *p* ≤ 0.001). 

Of the 915 patients who underwent spinal anesthesia, 27 women experienced side effects such as headaches in the frontal and occipital region of severe and moderate severity, as well as nausea and vomiting. Of the 27 women, these side effects were severe in 13 women and moderate in the remaining 14 patients. Thirteen patients who exhibited severe symptoms of headache, nausea, and vomiting underwent manipulation-plumbing. In addition, an additional group of 14 patients with moderate headache received drug correction, which encompassed the following approaches:

– The use of intravenous infusion solutions in the amount of 1 L;
– A total of 8 mg of Dexamethasone solution;
– Thereafter, use of subcutaneous application of a solution of caffeine sodium benzoate, 200 mg^−1^ mL every 8 h;
– Oral administration of paracetamol tablets, 500 mg every 8 h.

The dynamics of changes in the patient’s condition were assessed after 24 h of drug therapy. 

Conversion to intubation anesthesia was performed in one case (0.11%). The indication for surgical intervention of this patient was symptomatic uterine fibroids, vesico- and rectocele, and stress urine incontinence. A laparoscopic hysterectomy was performed under spinal anesthesia. After 2.5 h of anesthesia, the patient began to wake up, and intubation anesthesia was installed. Among 915 patients with SA, 31 (5.3%) concurrently needed time sedation and 17 (2.9%) intravenous narcotic analgesics—none of the 915 patients needed to be converted to GA with intubation because of nausea and vomiting. 

Among our 915 patients with laparoscopic surgery under spinal anesthesia, there were no respiratory arrest cases. The duration of laparoscopic surgeries using spinal anesthesia ranged between a minimum of 20 min and a maximum of 175 min, while the average was 47 min among 915 patients. The antalgic effect of spinal anesthesia (bupivacaine hydrochloride) lasted between 120 min to 240 min, including the postoperative time.

Two separate ANCOVAs were performed to assess the effect of the CO_2_ (mmHg) gas on enduring pain with weight as the covariate. In the first analysis, the effect was considered for obese patients versus patients with normal weights. It was found that every term of the model was statistically significant (*p* < 0.01) and that the amount of CO_2_ gas was the so-called effect modifier for experiencing pain. Thus, a higher amount of CO_2_ gas in obese patients indicated a higher chance of experiencing pain. For example, if the amount of CO_2_ gas was 8 mmHg then the odds ratio, that is, the chance of experiencing pain to the chance of being pain-free, was 1.6. This means that an obese patient is 60% more likely to experience pain during the surgery than a patient with a normal weight. The chance of experiencing pain in obese patients rapidly increased with a higher amount of CO_2_ gas, and for 10 mmHg of CO_2_ gas, an obese patient was more than 6 times, which is more than 600%, more likely to experience pain than a patient with a normal weight.

The second analysis pertained to the consideration of the effect of CO_2_ (mmHg) in controlling pain in underweight patients versus patients with normal weights. In this case, it was found that although not every term in the model was statistically significant, the interaction between the two variables was statistically significant at the 10% level (*p* = 0.08). Moreover, in this case, it was found that the break-even value for the CO_2_ gas was about 5.6 mmHg. This means that if the amount of the CO_2_ gas was higher than 5.6 mmHg, then a patient with a healthy weight was less likely to experience pain. On the other hand, if the amount of CO_2_ was less than 5.6 mmHg, then a patient with normal weight was more likely to experience pain. For example, if the amount of CO_2_ was 5 mmHg, a patient with normal weight was 35% more likely to experience pain (the odds ratio was 1.35), while for an amount of 7 mmHg of the gas, a patient with normal weight was about 48% less likely to feel pain during surgery (odds ratio was 0.52). Table 4 provides the calculated odds ratio for varying values of CO_2_ (mmHg) for both analyses. 

Table 5 presents data on the intraoperative side effects experienced by a total of 915 patients. Each row in the table corresponds to a specific side effect, and the columns provide information on the number of patients who experienced each side effect and the percentage of patients it represents. 

The first side effect listed in the table is “Pain”. Out of the 915 patients, 22 patients reported experiencing pain during the operation. This corresponds to a percentage of 2% of the total patient population. The second side effect is “Nausea”. Among the 915 patients, 48 patients reported experiencing nausea. This accounts for 5.2% of the total patient population. The third side effect is “Nausea and Vomitus” (vomiting). This refers to patients who experienced both nausea and vomiting during the procedure. Out of the 915 patients, 13 patients reported this combination of symptoms, which represents 1.4% of the total patient population. The final side effect listed in the table is “Pain, Nausea, and Vomitus”. This refers to patients who reported experiencing pain, nausea, and vomiting simultaneously during the operation. Among the 915 patients, 11 patients reported this combination of symptoms, accounting for 1.2% of the total patient population.

In summary, the table provides quantitative data on the occurrence of different intraoperative side effects, including pain, nausea, nausea and vomiting, and pain along with nausea and vomiting. The numbers and percentages allow for a scientific analysis of the prevalence of these side effects within the observed patient population.

## 4. Discussion

In the meantime, more than 3212 laparoscopic surgeries in the Trendelenburg position were performed in Urgench in collaboration with the clinical team of Switzerland [5,18]. It is, to our knowledge, the biggest number performed worldwide in gynecology. Intubation and intravenous anesthesia for such operations became history in Urgench, Uzbekistan, just like cesarean sections did. After a learning phase and becoming used to performing laparoscopic surgery using spinal anesthesia and the Trendelenburg position, the anesthetists, the surgeons, and the patients are not interested in performing such surgeries in GA with intubation anymore. In Urgench, spinal and peridural anesthesia for laparoscopic surgeries became the new standard of care for cesarean section.

The relationship between patient weight and pain during surgery can be complex and multifactorial. It is important to consider various factors that may contribute to this relationship, such as body composition, tolerance to pain, and individual physiological differences [9,11,13,14]. The results presented highlight the potential influence of patient weight on the experience of pain during surgery when CO_2_ is used. It suggests that overweight patients may have a higher chance of experiencing pain when the applied amount of CO_2_ exceeds a certain threshold. Regarding overweight patients, it is plausible that the increased amount of CO_2_ applied during surgery may result in a higher chance of pain exceeding a certain threshold. The excess weight and adipose tissue in overweight individuals can lead to increased intra-abdominal pressure, which may contribute to discomfort or pain during the insufflation of CO_2_. Additionally, the distribution of adipose tissue may affect the spread and dispersion of CO_2_, potentially leading to variations in pain perception [22,23,24]. Scheib et al. reviewed relevant studies of the existing literature on the relationship between patient weight, CO_2_ application, and pain during surgery [25]. The impact of patient weight on pain perception during laparoscopic surgery was examined. The authors described those overweight patients as experiencing higher levels of pain compared to normal-weight patients when the CO_2_ pressure exceeded a certain threshold. This finding was attributed to the increased intra-abdominal pressure resulting from excess adipose tissue in overweight individuals. 

In contrast, a study [26] focused on underweight individuals and their response to CO_2_ insufflation during laparoscopy. The results indicated that underweight patients were less likely to experience pain as the amount of CO_2_ pressure increased. The researchers hypothesized that the lower amount of adipose tissue in underweight individuals allowed for a more even distribution of CO_2_ pressure, reducing the likelihood of pain exceeding a certain threshold. In another study, however, [5] found no significant association between patient weight and pain during laparoscopic surgery and suggested that factors other than weight, such as individual pain thresholds and surgical technique, may have a more substantial impact on pain perception. Our results confirm that underweight individuals are less prone to pain with increasing CO_2_ pressure. 

It is important to also note that individual variations exist within each weight category, and other factors, such as overall health status and previous surgical experiences, can also influence pain perception during surgery [5]. Additionally, the discussion focuses solely on the influence of CO_2_ pressure on pain and does not consider other aspects that may contribute to pain during surgery, such as surgical techniques or individual pain thresholds. Further studies are required to better define the indications and contraindications of laparoscopic surgeries under loco-regional anesthesia.

In our experience of 915 patients, a good compromise is 8 mmHg or lower; sometimes 10 mmHg is also well endured. In the beginning, a surgeon has to take time to adapt to working with such low pressures. Such an adaptation, however, can occur quickly if the surgeon is motivated, and this can occur without respiration problems in the patient by doing such surgery with a pneumoperitoneum of 8 mmHg and in a Trendelenburg position of 30–45°. To have good laparoscopic visibility and not be disturbed by intestines, some precautions are important: first, a good preoperative preparation of the bowel is required; second, a Trendelenburg position of 30–45° should be employed; and third, manipulation of the uterus by a vaginal or laparoscopic device.

By using the described technique below and by monitoring and vigilance, there is a very low risk for the patient. In rare cases, however, complications may occur. Therefore, materials and devices for general anesthesia must be available for immediate use. It is important to have a good collaboration between the anesthesiologist and the gynecological surgeon. This concept may become routine, where the experience of performing a laparoscopy with low intraperitoneal pressure already exists, as is the case in many general hospitals or specialized minimal-invasive surgery units. Patient selection is another important aspect of laparoscopy success. Once the patients are adequately informed and offered the possibility of local anesthesia, many of them will opt to receive spinal anesthesia for the laparoscopic operation.

Loco-regional anesthesia for laparoscopic surgery using the Trendelenburg position is not only feasible but should be discussed as being used in many cases as the first choice due to the advantages it brings for the patients, as well as the feasibility and the need for special material used in general anesthesia. A special case is with obese patients, who have more pain with a higher CO_2_ pressure, which is needed for the visibility of the abdominal organs by the surgeon. It should also be considered that cardiovascular-related problems may be associated more often with obese patients. In these cases, careful consideration must be taken on which kind of anesthesia to choose. In rare pathologic cases, general anesthesia with intubation should remain the first choice. Pain is usually combined with high intra-abdominal pressure and occurs at the end of the operations, especially during long and extensive surgeries.

In this study, the inclusion and exclusion criteria for the study were checked at the preoperative consultation. The patient was informed about the different types of anesthesia, possible complications, and side effects, and about postoperative care. All patients were given time to reflect on the information provided and to sign informed consent. The surgeons, as well as postoperative nurses, were informed about which type of anesthesia would be applied. All specialists were trained before the introduction of the technique in the hospital, including junior staff. The patient group for surgery included patients with fertility problems, ovarian cysts of different sizes, and endometriosis. In terms of pain, patients experienced little to no pain during the surgical procedure, and if sensitivity increased, the patient immediately informed the anesthesiologist, and measures were taken to reduce the pain. Each patient underwent a postoperative questionnaire, including a pain assessment.

Moreover, loco-regional anesthesia could become the first choice of anesthesia for the new surgical vaginal approach, called the vNOTE (vaginal natural orifice transluminal endoscopic surgery) technique, which combines laparoscopic instrumentation and skills exclusively by the vaginal route for surgery of the uterus and adnexa [27]. It is an important advantage to perform this surgery in spinal or peridural anesthesia since abdominal trocars are not used, and therefore, anesthesia does not need to be effective in the abdominal area. Another advantage is that the vNOTE approach does not need a second surgical assistant moving the uterus since the main surgeon is moving the uterus with his vaginal endoscopic instruments. It also has to be mentioned that the endoscopic vaginal procedure uses low CO_2_ intra-abdominal pressure (8 mmHg) and carries a lower risk of side effects.

Intraoperative nausea and vomiting can be minimized through effective implementation of perioperative strategies [5,18]. Based on our accumulated expertise, the intravenous administration of specific compounds, such as Dexmedetomidine from EVER Pharma in Unterach, Austria, known for its psychotropic effects, has demonstrated noteworthy results in reducing side effects associated with spinal anesthesia and laparoscopic surgery, including pain, nausea, and vomiting [28,29,30]. Our recent findings indicate a significant decrease in the occurrence of these side effects to 2.9% (19 out of 658 patients) under medical treatment with Dexmedetomidine in contrast to the observed rate of 10.3% (94 out of 915 patients) without the use of Dexmedetomidine. We used Dexmedetomidine against nausea and vomitus in these additional 658 patients and will present the detailed results in the following paper. Even if vomiting occurs, it is not at risk for patients and is only slightly disturbing for a few minutes until the surgeon can restart his activity after the patient vomits. Since patients are awake or easily awakened, there is no danger of aspiration into the lungs. Dexmedetomidine is used for the sedation of adult surgical patients who require a depth of sedation that still allows awakening through verbal stimulation. It can be used for non-intubated patients before and/or during surgical procedures requiring procedural sedation/wax sedation. It acts additionally as a tranquilizer for individuals who suffer from anxiety and pain. It relieves stress and relaxes patients in cases of nervous tension. Compared to other drugs like midazolam and propofol, patients are generally more easily awakened and more cooperative and independent if they are in pain or not. As implemented in this study, larger sample sizes and standardized pain assessment tools enhanced the validity and generalizability of the findings. This shows that the results can be improved if the surgeon becomes used to working under lower intraperitoneal CO_2_ pressure and by using new compounds like Dexmedetomidine, decreasing significant side effects.

The available literature provides insights into the relationship between patient weight, CO_2_ application, and pain during surgery. Future studies should aim to address the limitations of existing research by considering additional factors that may influence pain perception and vomitus, such as body composition, tolerance to pain, and individual physiological differences. A limitation of our study was that the assessors of postoperative outcomes were not blinded to the type of anesthesia. Based on the results of our study on local anesthesia in gynecological surgery, a randomized clinical study is warranted.

## 5. Conclusions and Recommendations

We propose recommendations based on our experience of more than 3212 cases and the detailed results of over 900 clinical observations. This study can be used to make laparoscopic surgeries less invasive, along with updating the normative regulations and improving perspectives of laparoscopic surgeries under loco-regional anesthesia. The following are some of the points we want to emphasize for practical use.

1. Thanks to a special technique of spinal anesthesia (puncture of the spinal space at the L2–L4 level, immediate transfer of the patient to the Trendelenburg position to ensure anesthesia to the Th10–11 level) and low intra-abdominal pressure (below 8 mm Hg), a decrease in the frequency of intra- and postoperative pain could be observed.
2. The patient is conscious during the entire operation. This allows the patient to see their organs on the screen, observe the progress of the operation, ask the surgeon questions of interest to her or answer the questions of medical personnel, and also actively participate in decision-making.
3. A good view for the surgeon is provided by strengthening the Trendelenburg position (30–45 degrees, which is ensured by the use of advanced spinal anesthesia techniques) and choosing the region of insertion of small trocars lower in the abdomen (not higher than the umbilicus).
4. Using the Trendelenburg position, by mobilizing the intestines towards the diaphragm, it is possible to work in the small pelvis with low intra-abdominal insufflation, which, in turn, reduces postoperative pain, hyperbaria, PE, diaphragm irritation, and a feeling of lack of air.
5. Informing patients of which kind of diet to use a few days before surgery in order to diminish the secretion and accumulation of liquid in the stomach and, thereby, diminishing nausea and the risk of vomitus during surgery.
6. Using anti-emetic and psychotropic treatment with new compounds of drugs to diminish pain, nausea, and vomitus during surgery.

By focusing on the health of the patient, which remains an important parameter in surgery, loco-regional anesthesia should be better defined in such surgeries and routinely implemented. A main concern lies within obese patients; special consideration has to be taken because of the higher intraperitoneal CO_2_ pressure required for good visibility and, therefore, the potential for a higher probability of pain. In such special cases, general anesthesia with intubation shall remain the standard of care. For this, more studies are needed to better define obese patients in whom general anesthesia for laparoscopic surgery should be preferred. Our next clinical study and analysis will include this topic and will focus on our experiences of how to diminish side effects.

## Figures and Tables

**Table 1:** Birth delivery parameters in Khorezm region, Uzbekistan.

Annual Birth Rate	Cesarean Section	Regional Anesthesia	Intubation
40,896	8795 (21.5%)	8634 (98.1%) (peridural)	161 (1.9%)

Source: Khorezm Healthcare Department, 2022.

**Table 2:** Basal characteristics of patients comparing patients without pain to patients with pain during surgery.

		Group 1 No Pain *n* = 847	Group 2 Little/Middle Pain *n* = 68	*p*-Value *
1	Age of operation—years	29.3 ± 6.49	30.2 ± 6.29	0.2571 a
2	Body mass index—kg/m^2^	24.1 ± 3.72	25.4 ± 5.12	0.07664 a
3	Obesity BMI > 30	63 (7.44%)	13 (19.12%)	0.003 b
4	Comorbid Diseases			
5	Anemia	713 (84%)	60 (88%)	0.6093 b
6	Adhesions	18 (2.1%)	6 (8.8%)	0.0062 b
7	Hepatitis B	11 (2.2%)	2 (2.9%)	0.2508 b
8	Hepatitis C	19 (2.2%)	3 (4.4%)	0.2204 b
9	Parity	440 (52%)	32 (47%)	0.3726 b

* a—Mann–Whitney test; b—Fisher’s exact test.

**Table 3:** Characteristics causing increased pain.

			First Group			Second Group			*p*-Value
			*n* = 847	P (%)	m (±)	*n* = 68	P (%)	m (±)	
1	Abdominal status	I	4	0.47	0.236	3	4.41	2.49	*p* < 0.001
		II	241	28.45	1.55	7	10.29	3.69	
		III	578	68.24	1.60	55	80.88	4.77	
		IV	24	2.83	0.57	3	4.41	2.49	
2	Lengths of operation	>30	99	11.6	1.10	4	5.88	2.85	*p* < 0.001
		31–60	648	76.51	1.46	38	55.88	6.02	
		<60	100	11.81	1.11	26	38.24	5.89	
3	Bleeding	0–50	801	94.57	0.78	61	89.71	3.69	*p* < 0.001
		˃50	46	5.43	0.78	7	10.29	3.69	
4	BMI	>25	531	62.69	1.66	35	51.47	6.06	*p* = 0.003
		25–30	253	29.87	1.57	20	29.41	5.53	
		˃30	63	7.44	0.90	13	19.12	4.77	
5	mmHg	0–4	150	17.71	1.31	4	5.88	2.85	*p* = 0.013
		5–8	697	82.29	1.31	64	94.12	2.85	

P—Percentage.

**Table 4:** Effect of weight in the amount of CO_2_ (mmHg) pressure on pain.

CO_2_ (mmHg)	OR (Obese vs. Non-Obese)	OR (Healthy Weight vs. Underweight)
4	0.097881276	2.161062502
5	0.195538107	1.349858808
6	0.390627835	0.84315877
7	0.780359943	0.526660053
8	1.558930486	0.328966289
9	3.114286273	0.205481351
10	6.221431345	0.128349278

**Table 5:** Incidence of intraoperative side effects.

	Intraoperative Side Effects	Number of Patients’ Symptoms out of 915	%
1	Pain	22	2%
2	Nausea	48	5.2%
3	Nausea and vomitus	13	1.4%
4	Pain nausea and vomitus	11	1.2%

## Data Availability

All datasets presented in this study are included in the article. Further inquiries can be directed to the corresponding author.
